# Responses to concerns raised about publications don’t address the concerns raised

**DOI:** 10.1177/20494637231198757

**Published:** 2023-08-29

**Authors:** Mark J Bolland, Alison Avenell, Andrew Grey

**Affiliations:** 1 Department of Medicine, 1415 University of Auckland, Auckland, New Zealand; 2 Department of Endocrinology, ADHB, Auckland, New Zealand; 3 Health Services Research Unit, 1019 University of Aberdeen, Aberdeen, Scotland

**Keywords:** Musculoskeletal pain, randomised trials, publication integrity, journal responses, retraction

## Abstract

Recently in the Journal, Amanda Williams described her experience of raising concerns about a group of trials with “untrustworthy data”. We were inspired by the work of Williams and colleagues to examine these and other trials by the same research group. Similar to Williams, we found that the patterns of reported data differed from the patterns expected to arise from valid randomisation. We also identified a high proportion of reported baseline p-values for categorial variables that differed from independently calculated p-values. We reported these findings to the affected journals but none of the concerns were addressed and no action will be taken about the majority. Despite the large number of unresolved concerns about these trials, readers will be unaware of the issues, which seems entirely unsatisfactory.

The systematic review by Amanda Williams and colleagues^1,2^ highlighted concerns about publication integrity in 10 randomised controlled trials (RCTs) by Marco Monticone and colleagues, based in part on unexpected distributions of baseline variables following randomisation. We have used similar techniques in the past and so explored whether the observed distribution of baseline categorical variables was consistent with the expected distribution in 17 RCTs by Monticone and colleagues identified in a PubMed search that reported categorical baseline variables (which included all 10 of the RCTs assessed by Williams and colleagues). The key finding was that the frequency counts were much more similar than expected.^3^ Figure 1 shows that in the 17 trials there are many more variables that differed by one or two between groups (e.g. 10 in group 1, 11 in group (2) compared to the expected numbers, and conversely there are many fewer variables with a difference of four or more between groups than expected. Since valid randomisation is fundamental to the interpretation of RCTs, the inconsistency of the baseline data in these trials with the expected patterns from randomisation raises concerns about the validity of their results. In addition, 20% of reported *p*-values differed from *p*-values calculated using the reported summary statistics.^3^ As the reported and calculated *p*-values should be identical (unless there are missing data), these incorrect *p*-values indicate impossible data. Collectively, these findings support and extend the earlier review by Williams and colleagues.^1^ A response to those concerns^4^ has failed to address the majority of the issues raised.^5^

**Figure 1.**
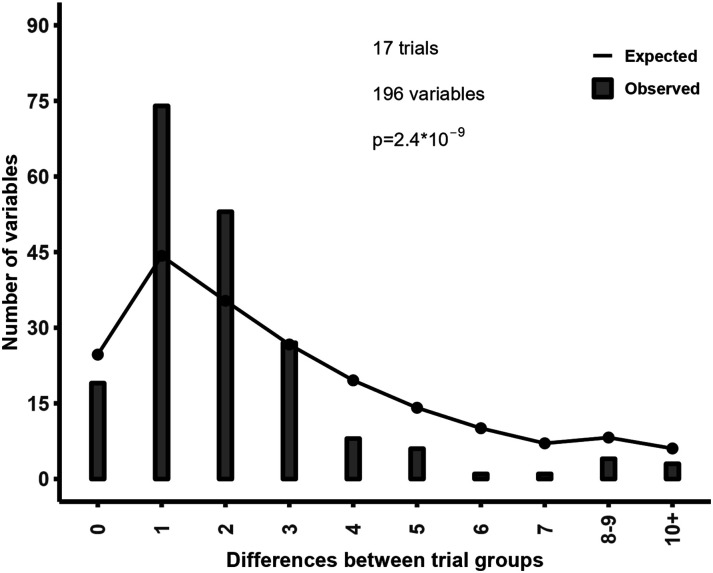
Observed and expected distribution of between-arm differences in frequency counts.

In March 2023, we emailed the six publishers of the eight journals that published the 14 unretracted RCTs together with the editor and/or journal contact. At that time, three of the 10 RCTs assessed by Williams and colleagues had been retracted. We received no response from one publisher or its journal; for two publishers, we received no response from the publisher but a response from the journal; and for three publishers, we received an acknowledgement or response from the publisher and the journal. Of the responses, one indicated that the paper had been retracted and a notice would shortly appear, although no notice has been published 4 months later. The publication has 162 citations in google scholar including 12 in 2023. Two responses reiterated that they have previously investigated – one indicated the new information was insufficient to investigate or request raw data, and the other did not respond when new specific information was provided. One treated the email as a request for advice about submitting a publication and failed to respond to the subsequent clarification. One said that they would not investigate because their RCTs were not referenced in our email and then requested an affirmation that the trials were problematic before they would investigate. During the email exchange, it became clear that some important information in an editorial about this issue was out of date (e.g. whether the articles had ethical approval or not), but the editor said it would not be updated. One response noted that one of their RCTs had been retracted and that they would consider what to do about the others. Finally, one indicated the journal had passed the issue to the publisher.

Thus, more than 4 months after notifying the journals and publishers, we understand that no action will be taken about eight RCTs, one will be retracted, decisions might still be in process about four articles, and for two we have no information. None of the RCTs have attracted an Expression of Concern. Our experience of notifying concerns about publication integrity in this case and others^6,7^ is similar to that of Williams.^2^ Despite the large number of unresolved concerns about the publication integrity of these RCTs, readers of the articles will be unaware of the issues, and potentially patients will be harmed if treatment decisions are based on unreliable trial results. This seems entirely unsatisfactory.

As Williams lays out clearly in her editorial,^2^ the tentacles of unreliable research spread far and wide, and there seems little appetite to bring about the changes necessary to remove unreliable articles from the literature.
